# Not All Overtime Is Equal: Differential Effects of Overtime Motivations on Employees’ Sustainable Consumption Behavior

**DOI:** 10.3390/bs16091521

**Published:** 2026-08-28

**Authors:** Zhiqing Zhang, Jiaqi Wang, Hao Li

**Affiliations:** 1 School of Economics and Management, Nanjing Tech University, Nanjing 211816, China; 2 College of Economics and Management, Nanjing University of Aeronautics and Astronautics, Nanjing 211106, China

**Keywords:** overtime motivation, sustainable consumption behavior, perceived control, family emotional support, organizational green climate

## Abstract

Employees increasingly experience blurred boundaries between work and private life, yet little is known about how workplace experiences influence consumer behavior beyond the workplace. Drawing on conservation of resources theory and ego depletion theory, and adopting the research perspective from the field of work–life spillover, this study employs survey data from 1030 Chinese employees to investigate the relationships among diverse overtime motives, perceived control, and sustainable consumption behavior, as well as the differences in these relationships within family and organizational contexts. The findings reveal that proactive overtime motivations (development-driven, subsistence-driven, and belonging-driven) are positively associated with green purchasing behavior, whereas reactive overtime motivations (escape-type, peer pressure, and mandatory) are negatively associated with reduced consumption behavior. Further analyses show that perceived control partially explains the positive relationship between proactive overtime motivations and green purchasing behavior, but does not mediate the associations between reactive overtime motivations and reduced consumption behavior. In addition, family emotional support strengthens compensatory consumption tendencies among employees experiencing involuntary overtime, whereas organizational green climate can strengthen the positive association between belonging-driven overtime motivation and green purchasing behavior. This study extends consumer behavior research by identifying overtime motivation as an important work-related correlate of sustainable consumption. The findings offer practical implications for managing overtime and fostering organizational green climates that support sustainable consumer behavior.

## 1. Introduction

Against the realistic background of carbon emission transfer under the dual-circulation new-development-pattern amid intensifying global climate change and growing pressure on natural resources, sustainable consumption has become central to the green transition and sustainable development (L. Chen et al., 2026). Sustainable consumption represents an ethical consumption paradigm in which consumers satisfy their basic living needs while taking intra-generational and inter-generational equity into account. It aims to reduce resource depletion and environmental load by optimizing consumption structure and restraining total consumption volume (Young et al., 2010; Lissillour et al., 2025). Typical everyday sustainable-consumption practices include purchasing energy-saving home appliances, organic fresh food, products with degradable packaging and recycled-fabric goods; buying food ingredients according to actual demand to avoid hoarding and spoilage; curbing impulsive consumption of digital products and fast-fashion items; and re-circulating idle goods and extending the service life of daily necessities. Recent United Nations Environment Programme (UNEP) evidence shows 1.05 billion tonnes of food were wasted at the household, food-service, and retail levels in 2022, and global e-waste reached a record 62 million tonnes in 2022, with only 22.3% formally collected and recycled (UNEP, 2024a, 2024b). The achievement of urban energy-climate resilience and high-quality development goals cannot be realized without the collaborative support of micro-level individual consumption behaviors. Accordingly, the environmental impacts of consumption have become an urgent governance issue (Wan et al., 2026a, 2026b; S. Chen et al., 2022; Rinot Levavi et al., 2025; Gomm et al., 2026).

The success of a consumption-side green transition ultimately depends on consumers’ decisions and choices (Ryghaug & Skjølsvold, 2023; Ollitervo et al., 2025). Employees occupy a particularly important position in the broader consumption landscape. As both creators of social wealth and primary decision-makers in household consumption, they are a key force in the sustainable consumption market and an important population through which to examine spillover from the work–life interface. Because work and nonwork domains are increasingly intertwined, employees’ environmental decisions may be systematically shaped by their work contexts (Composto, 2025; Trivedi et al., 2025). However, employees’ pro-environmental consumption choices do not emerge in isolation; their decision-making processes are constrained by the state of psychological resources derived from the workplace (X. Han et al., 2025). According to conservation of resources theory, individuals possess an innate tendency to actively accumulate and safeguard various physical and psychological resources. Sufficient resource reserves can underpin sustainable-consumption decisions that take long-term environmental benefits into account (Hobfoll, 1989; Sonnentag & Meier, 2024). Ego depletion theory further suggests that sustainable consumption demands constant self-restraint and rational trade-offs, which continuously deplete limited self-control resources. When psychological resources are persistently drained, individuals tend to opt for immediate gratification and voluntarily abandon pro-environmental consumption behaviors (Baumeister et al., 1998; Daiss et al., 2025).

As a typical workplace stressor, overtime may influence employees’ sustainable consumption behavior by altering individuals’ resource reserves. In the contemporary workplace, overtime has become a universal phenomenon across countries and development stages. OECD (2026) data indicate that workers in OECD countries worked approximately 1736 h per year in 2025 (approximately 33 h per week), with annual working hours exceeding 2000 in some countries. Overtime is particularly prevalent in China. In December 2025, employees in Chinese enterprises worked an average of 48.6 h per week, substantially above the statutory limit of 40 h (National Bureau of Statistics of China, 2026). The pressures associated with overtime increasingly spill over into employees’ nonwork lives. Existing research has focused primarily on the adverse effects of overtime on physical health, psychological well-being, emotions, and work-related outcomes (Kivimäki et al., 2015), while paying less attention to cross-domain spillover. In particular, the effects of overtime on sustainable consumption behavior, which requires deliberation, self-control, and long-term responsibility, remain insufficiently understood.

From behavioral science and cross-domain spillover perspectives, overtime may shape employees’ consumption and ethical decisions through the dual mechanisms of resource depletion and psychological compensation (Shen et al., 2025). Ego depletion theory suggests that the cognitive burden imposed by overtime impairs self-regulation (Kapoor & Tripathi, 2020), increasing preferences for immediate gratification and thereby reducing sustainable consumption behaviors that require long-term self-discipline. At the same time, the loss of autonomy associated with overtime may lead individuals to seek emotional compensation through consumption (Kilroy et al., 2020), which conflicts with the rationality, altruism, and long-term responsibility underlying sustainable consumption. Prior studies have largely emphasized macro-level values or individual traits as antecedents of consumption (Farrow et al., 2017). Consequently, the cross-domain link between overtime and consumption behavior remains underexplored, and systematic theory and empirical evidence concerning the psychological pathways through which overtime motivations affect sustainable consumption are particularly limited.

Against this background, this study draws on work–life spillover perspectives and survey data from 1030 employees to examine how overtime motivations affect employees’ sustainable consumption behavior and to clarify the underlying mechanisms and boundary conditions. This study makes three theoretical contributions. First, it reconceptualizes overtime as a motivationally differentiated work experience rather than a homogeneous job demand. By distinguishing proactive and reactive overtime motivations, the study explains why working beyond regular hours may have divergent implications for employees’ private consumption. Second, it refines the cross-domain analytical framework for work–life interface by identifying perceived control as a deliberative mechanism through which proactive overtime motivations are linked to green purchasing behavior, while showing that reactive overtime motivations are more closely associated with restraint-related consumption outcomes outside this control-based pathway. Third, it contributes to sustainable consumption research by differentiating substitution-oriented green purchasing from restraint-oriented reduced consumption. This distinction reveals that sustainable consumption behaviors are not equally sensitive to work-derived resources and motivational strain.

## 2. Literature Review

### 2.1. Sustainable Consumption Behavior and Overtime Motivations

Sustainable consumption behavior is commonly understood as a set of pro-environmental and ethically informed practices through which consumers satisfy personal needs while reducing ecological harm and resource waste (Barone et al., 2024). In employee populations, two behavioral expressions are especially relevant: green purchasing behavior and reduced consumption behavior (Andrade & Vieites, 2025). Green purchasing refers to choosing products and services with superior environmental attributes, often requiring information search, evaluation of environmental claims, and acceptance of possible price premiums. Reduced consumption, by contrast, involves limiting unnecessary purchases, avoiding waste, and exercising restraint in response to immediate desires (Joanes et al., 2020; Gatersleben et al., 2002; Munaro et al., 2024). Although both behaviors contribute to sustainable consumption, they are not psychologically identical. Green purchasing is primarily substitution-oriented and depends on deliberation and positive outcome expectations, whereas reduced consumption is restraint-oriented and requires sustained self-regulation. This distinction is important because work experiences may supply or drain the psychological resources needed for each form of sustainable behavior.

The enactment of individual sustainable consumption is highly dependent on the supply of psychological resources. As a prevalent workplace resource-disturbing event, overtime can alter employees’ resource gain or depletion state due to differences in its underlying motives, thereby exerting differential effects on the two types of consumption behaviors (Hollebeek et al., 2023; Drago et al., 2009). Overtime motivations refer to the reasons employees work beyond regular hours and capture the subjective meaning attached to overtime. Earlier studies often distinguish overtime as voluntary versus involuntary or as intrinsically versus extrinsically motivated, but such binary classifications may obscure the heterogeneous motives through which overtime is experienced.

Drawing on the core logic of distinguishing resource gain and resource depletion in conservation of resources theory and the work-context characteristics of Chinese employees, this study distinguishes six overtime motives: development-driven, subsistence-driven, belonging-driven, escape-type, peer pressure, and mandatory overtime. The first three motivations are proactive, stemming from employees’ aspirations for personal growth, needs for economic security, or emotional identification with their organization (Amabile et al., 1994; Avey et al., 2009). By contrast, the latter three reactive overtime motivations are all triggered by external forces or negative psychological demands. Among them, escape-type overtime refers to the practice in which employees treat overtime as a way to get away from adverse daily-life scenarios. Specifically, employees work overtime to escape tedious housework, family responsibilities of childcare, cumbersome domestic routines and meaningless social engagements, so as to evade the distress and pressure arising from daily life (Liu et al., 2022). Peer-pressure overtime arises from the coercive peer atmosphere in the workplace (Schaufeli et al., 2006; Beckers et al., 2008), whereas mandatory overtime results from explicit arrangements imposed by supervisors (Beckers et al., 2008; Golden & Wiens-Tuers, 2005). The classification system of overtime motivations adopted in this study takes individuals’ internal subjective attributions when engaging in overtime work as the core criterion, rather than external objective contextual factors such as overtime hours or personal schedule arrangements. As situated psychological perceptions, motivations are not stable inherent traits attached to individual employees. Faced with different overtime scenarios, the same worker may form distinct subjective interpretations based on the immediate triggers of overtime, which can be categorized into different motivation types accordingly. This distinction allows overtime to be examined not merely as an extension of working time but as a motivationally differentiated work experience that may spill over into nonwork consumption.

Conservation of resources theory and ego depletion theory provide a useful foundation for linking overtime motivation to sustainable consumption. Conservation of resources theory argues that individuals strive to acquire, conserve, and protect valued resources, including time, energy, money, and psychological capacity (Hobfoll, 1989). Ego depletion theory further suggests that self-control and deliberative choice rely on limited psychological resources, and that depletion can impair restraint and long-term decision-making (Vohs & Faber, 2007). From these perspectives, the behavioral consequences of overtime should depend not only on the number of hours worked but also on whether the overtime is appraised as a resource investment or as a resource loss. Because green purchasing and reduced consumption require different configurations of deliberation, autonomy, and self-regulation, distinct overtime motivations may be associated with these behaviors in different ways.

Proactively motivated overtime may be linked to sustainable consumption through resource acquisition and positive work-related meaning. Development-driven overtime can provide opportunities for competence building and mastery, which may support cognitive engagement and careful information processing (Dong et al., 2025). Subsistence-driven overtime can increase financial security and reduce sensitivity to the costs often associated with greener products (Babutsidze & Chai, 2025). Belonging-driven overtime may strengthen social connection and value consistency by reinforcing employees’ identification with their organization (Divya & Christopher, 2024). These resource and meaning pathways are particularly relevant to green purchasing, which requires consumers to evaluate product attributes and align purchases with longer-term values (Schofield & Venkataramani, 2021). At the same time, the implications of proactive overtime for reduced consumption may be less direct, because restraint-oriented behavior depends primarily on inhibiting desire rather than expanding consumption options.

Reactive overtime, by contrast, is more likely to be experienced as externally imposed demand. Escape-type, peer-pressure, and mandatory overtime can reduce time autonomy, increase fatigue, and consume emotional and cognitive resources without providing equivalent compensatory gains (Dong et al., 2025). Such conditions may weaken self-control and make restraint-oriented behaviors, such as reduced consumption, more difficult to sustain (Kapoor & Tripathi, 2020). Resource depletion may also limit the information search and deliberative evaluation required for green purchasing, although its influence on purchasing choices may depend on additional norms, identities, and contextual cues. Taken together, the literature suggests that different overtime motivations may have distinct associations with the two dimensions of sustainable consumption. Accordingly, this study proposes the following hypotheses:

**H1.**  *Different overtime motivations exert differential effects on sustainable consumption behavior.*

**H1a.**  *Proactive overtime motivations (development-driven, subsistence-driven, belonging-driven) are positively associated with sustainable consumption behavior (reduced consumption behavior, green purchasing behavior).*

**H1b.**  *Reactive overtime motivations (escape-type, peer-pressure, mandatory overtime) are negatively associated with sustainable consumption behavior (reduced consumption behavior, green purchasing behavior).*

### 2.2. The Mediating Role of Perceived Control

Perceived control refers to individuals’ belief that they can influence relevant events, choices, and outcomes. It is worth noting that perceived control differs conceptually from locus of control, a classic psychological construct. The locus of control proposed by Rotter (1966) represents a stable personality trait, reflecting an enduring and relatively immutable attributional tendency that centers on whether individuals attribute event outcomes to internal efforts or external factors. By contrast, perceived control in this study is a situated psychological state without the property of dispositional stability. It specifically refers to employees’ immediate subjective judgment of mastery over time allocation, financial budgeting, emotion regulation, pro-environmental actions, and the effectiveness of environmental improvement within their current work and consumption contexts. Although both constructs revolve around mastery-related beliefs, they differ fundamentally in construct level, stability, and applicable contexts. It is conceptually close to self-efficacy but places stronger emphasis on subjective control over circumstances and behavioral consequences (Bandura & Wessels, 1997). In the consumer and environmental behavior research, perceived control is important because sustainable choices often require planning, confidence in one’s ability to act, and belief that individual behavior can make a difference. Higher perceived control can support deliberate decision-making and behavioral consistency, whereas lower perceived control is associated with stress, short-term coping, and weaker self-regulation (Fielder et al., 2026; Tan et al., 2025).

Overtime motivations may shape perceived control by altering employees’ access to valued resources. From a conservation-of-resources perspective, proactive overtime can be interpreted as an investment that generates competence, income, social recognition, or identity affirmation, all of which may strengthen employees’ sense of control over work and nonwork decisions (M. Han et al., 2024). Reactive overtime, however, is more likely to be interpreted as a demand that restricts time autonomy and drains psychological resources. When individuals are continuously exposed to such depleting demands without compensatory resource replenishment, their sense of personal mastery tends to be undermined (Bakker & Demerouti, 2007).

Perceived control may therefore serve as a psychological mechanism that channels long-term motivations into sustainable consumption behavior. Employees who feel greater control over time, finances, emotions, behavior, and outcomes should be better able to make consumption decisions that reflect long-term values rather than short-term pressure (Ten Brummelhuis & Bakker, 2012; Bakker & Demerouti, 2017). For green purchasing, perceived control can increase confidence in evaluating alternatives and acting on environmental preferences. For reduced consumption, perceived control may support the restraint needed to resist unnecessary purchases. On this basis, the following hypotheses are proposed:

**H2.**  *Perceived control mediates the relationship between overtime motivations and sustainable consumption behavior.*

**H2a.**  *Proactive overtime motivations enhance perceived control and thereby exert a positive effect on sustainable consumption behavior.*

**H2b.**  *Reactive overtime motivations reduce perceived control and thereby exert a negative effect on sustainable consumption behavior.*

### 2.3. The Moderating Roles of Family Emotional Support and Organizational Green Climate

Behavioral decisions are embedded in social contexts; therefore, the relationship between overtime motivations and sustainable consumption is unlikely to depend solely on individual psychological resources (J. Wang et al., 2026). Work–family and organizational climate research indicates that proximal social environments influence how employees interpret work experiences and transmit them to nonwork behavior. The family and the organization are particularly important because they provide emotional resources, behavioral norms, and value cues that may either strengthen or weaken the spillover from work to consumption (Zafar et al., 2025). Accordingly, this study considers family emotional support and organizational green climate as contextual moderators in the overtime motivation–sustainable consumption relationship.

Family emotional support refers to the emotional bonds, mutual care, and constructive communication among family members. Prior research suggests that family support can serve as a resource reservoir by helping employees recover from work-related strain and maintain psychological resilience (Ten Brummelhuis & Bakker, 2012; Sonnentag & Fritz, 2015). From the perspective of conservation of resources theory, family companionship and emotional care constitute core social resources for individuals. Overtime directly crowds out family time, substantially reduces positive emotional exchanges, induces the erosion of family emotional resources and fuels anxiety about resource loss. As a result, individuals become more likely to pursue material and emotional compensation via consumption (Hollebeek et al., 2023). Drawing on ego depletion theory, sustaining sustainable and restrained consumption choices actively consumes finite self-control resources. Negative emotions arising from insufficient family emotional bonds aggravate ego depletion, impair individuals’ psychological capacity to curb impulsive consumption, and consequently increase the probability of compensatory consumption behaviors (Baumeister, 2003; Cao et al., 2025). Supportive family relationships may help employees cope with the strain of overtime and maintain self-regulation. However, on the other hand, when employees experience stronger emotional support and fulfillment in family life, they may place greater value on nonwork time and family interaction. Under conditions of externally imposed overtime, such as peer-pressure or mandatory overtime, the loss of time that could otherwise be spent in an emotionally rewarding family context may be perceived as especially salient. This perceived deprivation may intensify frustration or guilt and increase the likelihood of compensatory consumption, thereby weakening reduced consumption behavior.

Organizational green climate refers to the extent to which an organization emphasizes environmental protection through its policies, practices, and shared norms (Zafar et al., 2025). Such a climate provides employees with cues about desirable environmental behavior and can encourage pro-environmental actions beyond formal job requirements (D. Wang et al., 2025). Meanwhile, individuals’ inherent environmental preferences also shape their career choices. Workers with stronger environmental tendencies are more likely to enter enterprises with a prominent green culture. The long-term aggregation of individual employees’ values ultimately contributes to the formation of the overall organizational green climate (Hicklenton et al., 2021). Grounded in the cross-domain logic of workplace experience spillover into personal consumption, this study focuses primarily on the influence of organizational green climate on individuals’ environmental cognition and thus excludes personal environmental values from the research model. Prior research also suggests that workplace environmental values may spill over into private life when employees internalize organizational norms and incorporate them into their self-concept (Kühner et al., 2025). This mechanism should be especially relevant for employees whose overtime is driven by organizational belonging, because their identification with the organization may make them more receptive to organizational environmental values (Ashforth & Mael, 1989; Maki et al., 2019). Thus, organizational green climate may strengthen the association between overtime motivations and sustainable consumption behavior. Accordingly, this study proposes the following hypotheses:

**H3.**  *Family emotional support moderates the relationship between reactive overtime motivations (escape-type, peer-pressure, mandatory overtime) and sustainable consumption behavior. The higher the level of family emotional support, the stronger the negative effect of reactive overtime motivations on sustainable consumption behavior.*

**H4.**  *Organizational green climate moderates the relationship between proactive overtime motivations (development-driven, subsistence-driven, belonging-driven) and sustainable consumption behavior. The stronger the organizational green climate, the stronger the positive effect of proactive overtime motivations on sustainable consumption behavior.*

**Figure 1:**
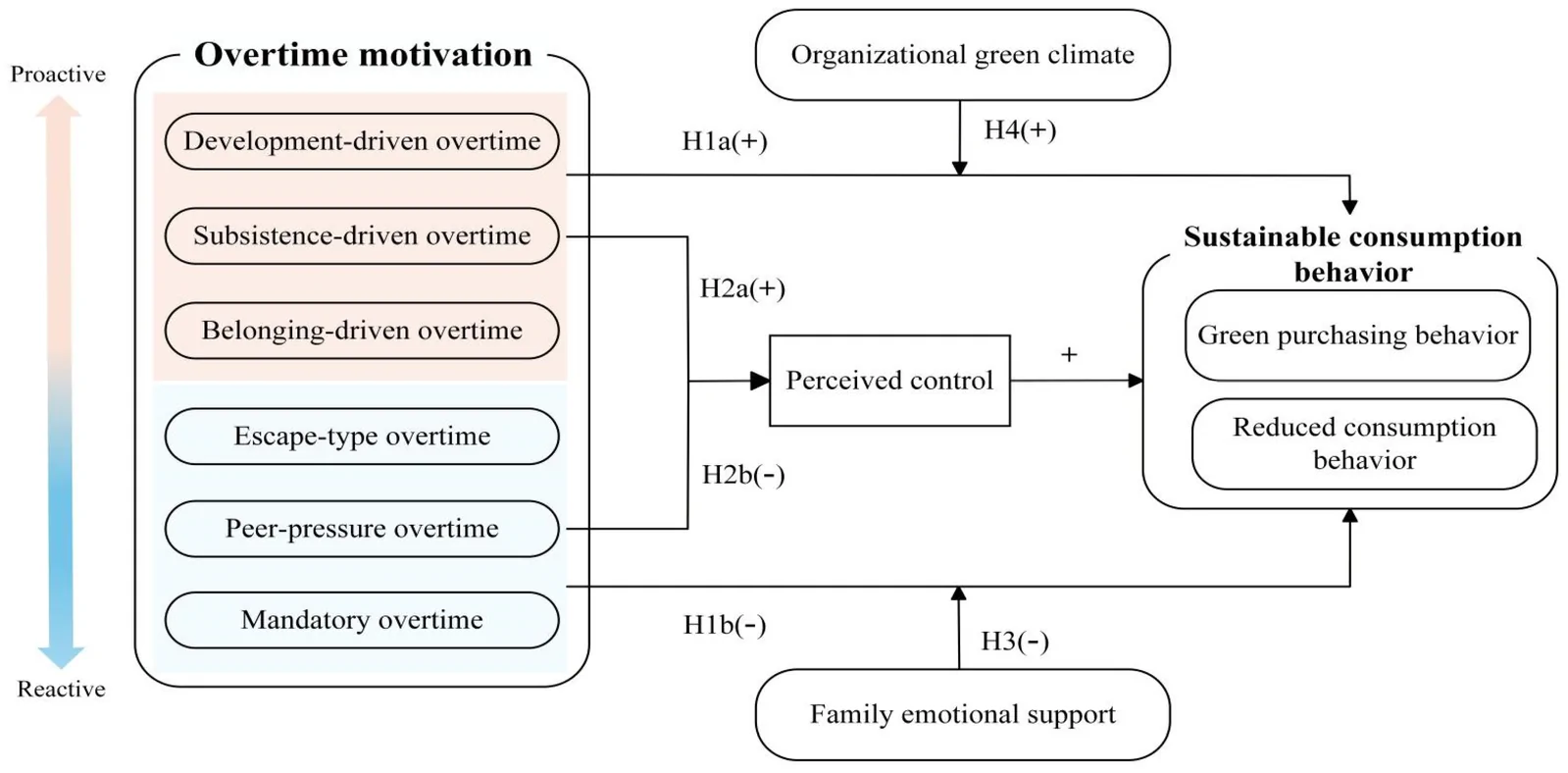
Conceptual model.

The conceptual model developed from the preceding analysis is presented in Figure 1.

## 3. Data and Methods

### 3.1. Variable Selection and Measurement

#### 3.1.1. Explained Variable: Sustainable Consumption Behavior

Sustainable consumption behavior is pro-environmental and ethically oriented behavior through which individuals balance personal needs, social development, and environmental protection while seeking to reduce resource waste and adverse environmental impacts. Its enactment depends heavily on deliberation, self-control, and long-term planning. Drawing on established sustainable consumption frameworks and the consumption characteristics of employees, this study distinguishes between reduced consumption behavior and green purchasing behavior, which respectively capture the scale and structure of consumption. These categories encompass the two principal pathways of everyday sustainable consumption: reducing unnecessary consumption and improving the environmental quality of consumption choices (Gatersleben et al., 2002; Joanes et al., 2020).

Reduced consumption behavior was operationalized by reverse-coding compensatory and non-essential consumption tendencies. The scale was adapted from Joanes et al. (2020). All reverse-scored items were reversely processed before analysis. Green purchasing behavior was measured using a scale adapted from Gatersleben et al. (2002). The measurement items are reported in Supplementary Materials Table S1.

#### 3.1.2. Explanatory Variables: Overtime Motivations

Previous studies of overtime motivation have generally adopted binary distinctions such as voluntary versus involuntary or intrinsic versus extrinsic motivation (Beckers et al., 2008). Although these distinctions capture the degree of autonomy involved in overtime, they do not fully reveal the different psychological mechanisms associated with specific motivational content. Some studies have further distinguished motives such as personal development, economic reward, organizational norms, and external coercion (Liu et al., 2022), but these dimensions may not fully capture features of the Chinese workplace, including strong organizational identification, conformity-based overtime, implicit coercion, and overtime used to escape pressures outside work. Drawing on multidimensional approaches to overtime motivation and the work-context characteristics of Chinese employees, this study adapts and extends existing classifications to identify six motives: development-driven, subsistence-driven, belonging-driven, escape-type, peer-pressure, and mandatory overtime. This classification permits a more context-sensitive examination of the heterogeneous mechanisms associated with overtime.

Both development-driven overtime and subsistence-driven overtime were measured based on the scale of Amabile et al. (1994). Among them, development-driven overtime corresponds to the intrinsic motivation dimension, referring to overtime behavior driven by intrinsic needs such as personal growth, ability improvement and work challenge. Subsistence-driven overtime corresponds to the extrinsic reward motivation dimension, referring to overtime motivation generated to obtain economic income and alleviate financial and employment pressure. Belonging-driven overtime refers to overtime motivation generated due to organizational belonging and emotional identification (Avey et al., 2009), and the scale items are derived from mature organizational identification scales.

Escape-type overtime is based on the definition and measurement tools of reactive overtime motivations in Liu et al. (2022), referring to overtime motivation generated to escape life pressure, social situations and so on. Peer-pressure overtime is based on the social influence and conformity overtime theory of Schaufeli et al. (2006) and Beckers et al. (2008), referring to overtime motivation generated due to colleague conformity and workplace atmosphere pressure. Mandatory overtime is based on the definition and measurement tools of mandatory overtime in Beckers et al. (2008) and Golden and Wiens-Tuers (2005), referring to reactive overtime motivation generated due to superior orders and organizational mandatory requirements.

The measurement items are reported in Supplementary Materials Table S1.

#### 3.1.3. Mediating Variable: Perceived Control

Existing studies show that perceived control includes control beliefs over different fields and objects, and a single dimension cannot fully reflect individuals’ control experience in consumption decisions (Pallant, 2000). Meanwhile, sustainable consumption has multiple attributes such as time input, economic cost, emotional regulation, behavior implementation and environmental effect judgment, which need to be explained by multi-dimensional sense of control (Bamberg & Möser, 2007; Vohs & Faber, 2007). Based on this, following the logic of “the whole process of consumption decision-making”, this study selects dimensions from five key links: time allocation, financial allocation, emotional regulation, behavior implementation and effect expectation, forming a complete measurement system covering “resources-psychology-behavior-outcome” to ensure high adaptation between dimensions and dependent variables. Finally, this study divides perceived control into five dimensions: time, finance, psychology, behavior and outcome. The five dimensions were defined and measured as follows.

Perceived control of time (Valcour, 2007) captures individuals’ subjective control over daily time allocation, rest, and vacation. Perceived control of finances (Burton et al., 1998) captures control over personal finances and consumption budgets. Perceived psychological control (Pallant, 2000) captures the ability to regulate emotional states and psychological experiences. Perceived behavioral control (Parker et al., 1995) captures the perceived ease and autonomy of engaging in environmentally friendly consumption. Perceived outcome control (Roberts, 1996) captures the belief that one’s consumption behavior can contribute to environmental improvement. The measurement items are reported in Supplementary Materials Table S1.

#### 3.1.4. Moderating Variables: Family Emotional Support and Organizational Green Climate

Family emotional support was measured as employees’ perceived warmth, care, understanding, and emotional fulfillment in everyday family life. The scale was adapted from Moos and Moos (2002), which captured the extent to which employees felt loved and cared for by family members, perceived the family as a warm and comforting environment, and experienced emotional fulfillment through family interactions. Organizational green climate was measured using the five-item scale developed by Dumont et al. (2017), which assesses the emphasis placed on environmental values, policy support, and environmentally oriented practices within the organization. The measurement items are reported in Supplementary Materials Table S1.

#### 3.1.5. Demographic Variables

This study draws on Kossek et al. (2026), Lange and Dewitte (2019), Neves et al. (2025), and Wiedmann et al. (2020), and ultimately selects gender, age, educational attainment, and household income as control variables. A meta-analysis by Kossek et al. (2026) from a life-cycle perspective reveals that employees of different ages and genders differ in disposable time resources and autonomy over arranging domestic affairs. Lange and Dewitte (2019) and Neves et al. (2025) argue that educational attainment shapes individuals’ propensity for pro-environmental consumption behaviors. Regarding household income, Neves et al. (2025) propose that higher household income enables individuals to better afford the price premium of green products, whereas Wiedmann et al. (2020) note that the association between income and pro-environmental behavior is not a simple positive correspondence and that this relationship operates through complex mechanisms.

### 3.2. Data Collection

Before the formal investigation, this study completed the systematic improvement of the scale. All measurement scales were adapted on the basis of relevant theories and combined with the actual context of overtime and consumption in the Chinese workplace, and a pre-investigation was carried out after review and optimization by experts in the field of organizational behavior. A total of 235 pre-investigation questionnaires were distributed through online channels, and 204 valid questionnaires were obtained after screening according to standards such as response time and logical consistency. After reliability and validity analysis, the first item of the peer-pressure overtime dimension was deleted, and a formal investigation scale with good adaptability was finally formed.

The formal survey was conducted through the Wenjuanxing platform between October and November 2025. Wenjuanxing is a widely used online research tool for empirical research in Chinese social sciences, and supports targeted sampling and multiple data-quality checks (J. Wang et al., 2026). This study defines employees specifically as workers with a formal employment contract with firms or organizations, excluding freelancers without a fixed employer. The prescreening questions at the start of the questionnaire directly eliminate respondents who are freelancers or have no regular overtime experience. To ensure that the research objects meet the set scope, the investigation adopted limited conditional sampling, only for in-service employees with actual overtime experience. Strict screening items were set at the beginning of the questionnaire: if the respondents were students, unemployed/unemployed, or had 0 weekly overtime hours in the past six months, they would directly terminate the answer and could not enter the formal questions, ensuring that all samples were target groups meeting the research requirements from the source. In the sampling process, demographic characteristics such as region, industry, enterprise nature and post type were evenly covered through the platform matching function to improve the representativeness and extensiveness of the sample as much as possible. After data collection, responses were retained only if completion time was at least 90 s, attention checks were passed, no duplicate IP address was detected, and no clear logical inconsistency was present. Of the 1250 questionnaires returned, 1030 were retained, yielding a valid response rate of 82.4%. The resulting sample was broadly distributed across demographic and employment categories, helping to reduce concerns about selection bias.

The valid sample was relatively young and highly educated. Respondents were concentrated in the 35–39-year age group, more than 70% held at least a bachelor’s degree, monthly income was most commonly between CNY 5001 and CNY 10,000, and the gender distribution was relatively balanced (54.4% women). Although younger employees were overrepresented, this pattern is consistent with the Chinese labor market, in which younger employees face intense career competition and financial pressure and tend to report higher overtime participation and longer overtime hours than older employees (National Bureau of Statistics of China, 2026). The sample also covered a wide range of industries and organizations and was reasonably distributed by gender, age, income, occupation, ownership type, and organizational size. Moreover, the screening procedure ensured that every respondent was currently employed and had recent overtime experience. Overall, the sample provided broad coverage of the target population and an adequate basis for the empirical analyses. Detailed characteristics are reported in Table 1.

### 3.3. Descriptive Analysis

#### 3.3.1. Overtime Status of Respondents

**Figure 2:**
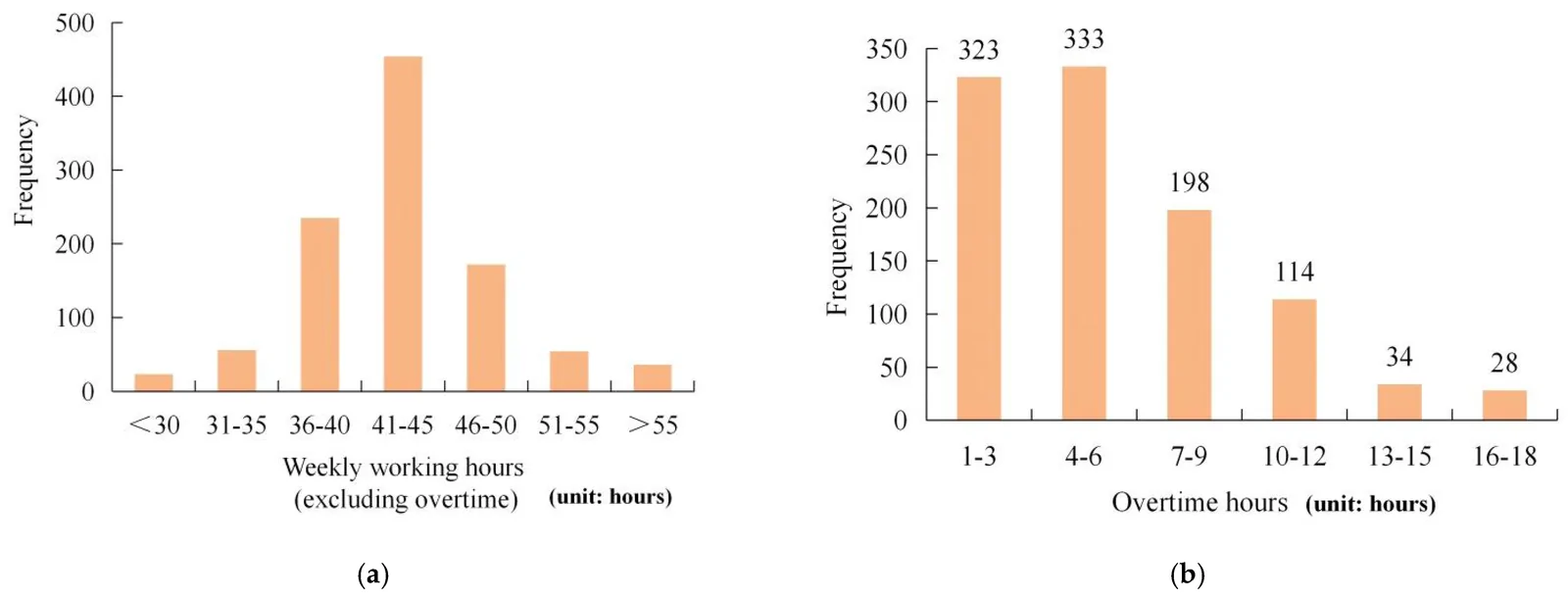

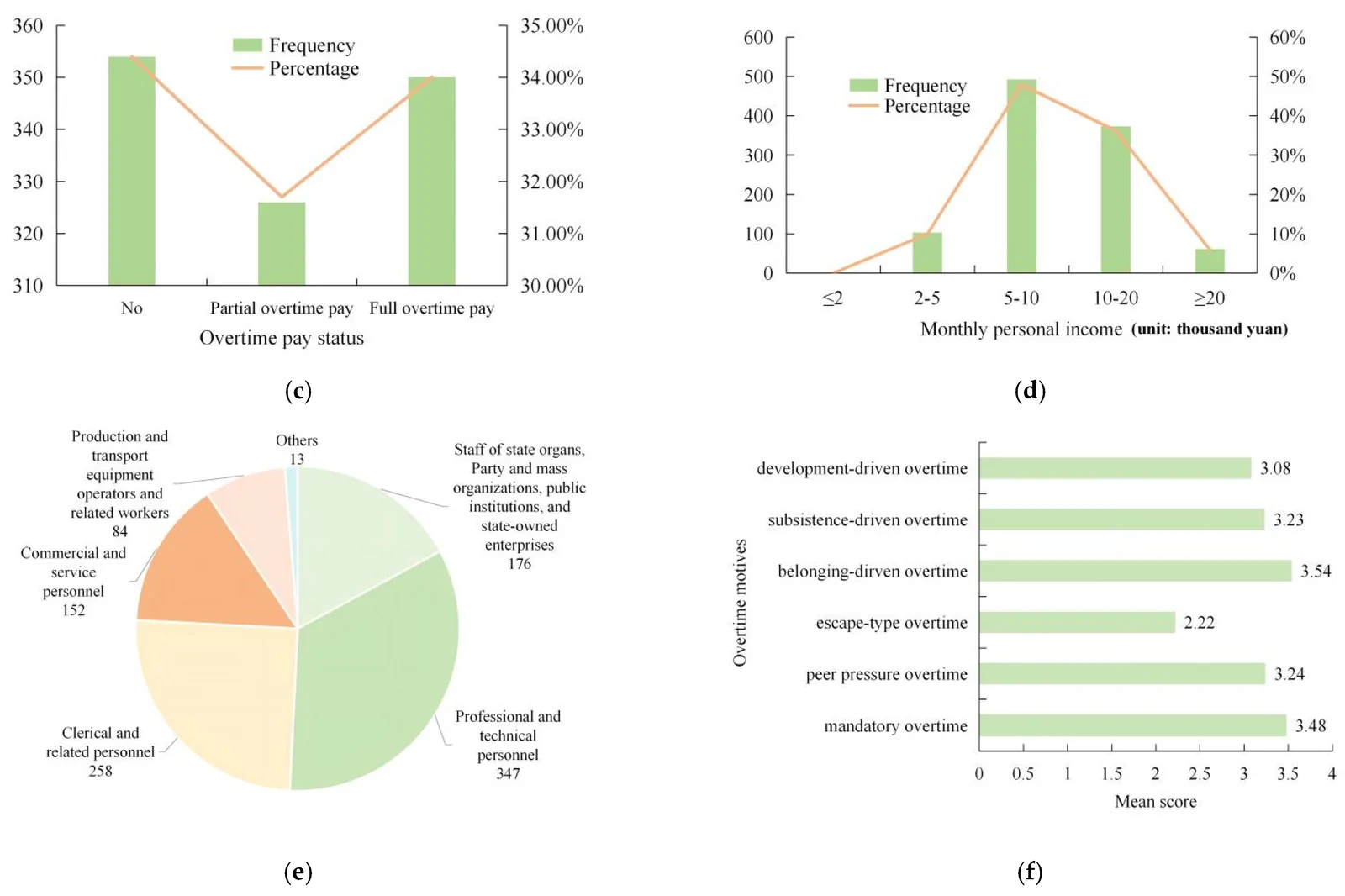
Distribution of overtime characteristics. Note: (**a**) shows the distribution of weekly regular working hours excluding overtime. (**b**) shows the distribution of respondents’ overtime hours. (**c**) shows the distribution of overtime pay status in the sample. (**d**) shows the distribution of respondents’ monthly personal income. (**e**) shows the occupational distribution of the sample. (**f**) shows the score distribution of six types of overtime motives.

As shown in Figure 2a, respondents reported an average weekly working duration (excluding overtime) of 42.78 h. Figure 2b shows that the sample’s average weekly overtime hours were 5.91 h, yielding a total average working time of 48.68 h for respondents. These data indicate that overtime is prevalent in the sample, consistent with findings from Shen et al. (2025) on overtime among Chinese employees. As illustrated in Figure 2c, compensation data further reveal the complexity of overtime arrangements: 34.3% of respondents received no overtime pay, and 31.6% received only partial compensation. More than 65% of overtime work was not fully remunerated, may be consistent with a substantial share of overtime is mandatory or implicitly coerced; this pattern also aligns with the conclusions of Shen et al. (2025).

From the mean scores of overtime motivations presented in Figure 2f, the proactive overtime motivations are ranked as belonging-driven (M = 3.54), subsistence-driven (M = 3.23), and development-driven (M = 3.08). For reactive overtime motivations, the ranking is mandatory overtime (M = 3.48), peer-pressure-driven overtime (M = 3.24), and escape-type overtime (M = 2.22). Overall, belonging-driven overtime, mandatory overtime, and peer-pressure-driven overtime are the three most prominent overtime motivations in this sample.

#### 3.3.2. Perceived Control and Sustainable Consumption of Respondents

**Figure 3:**
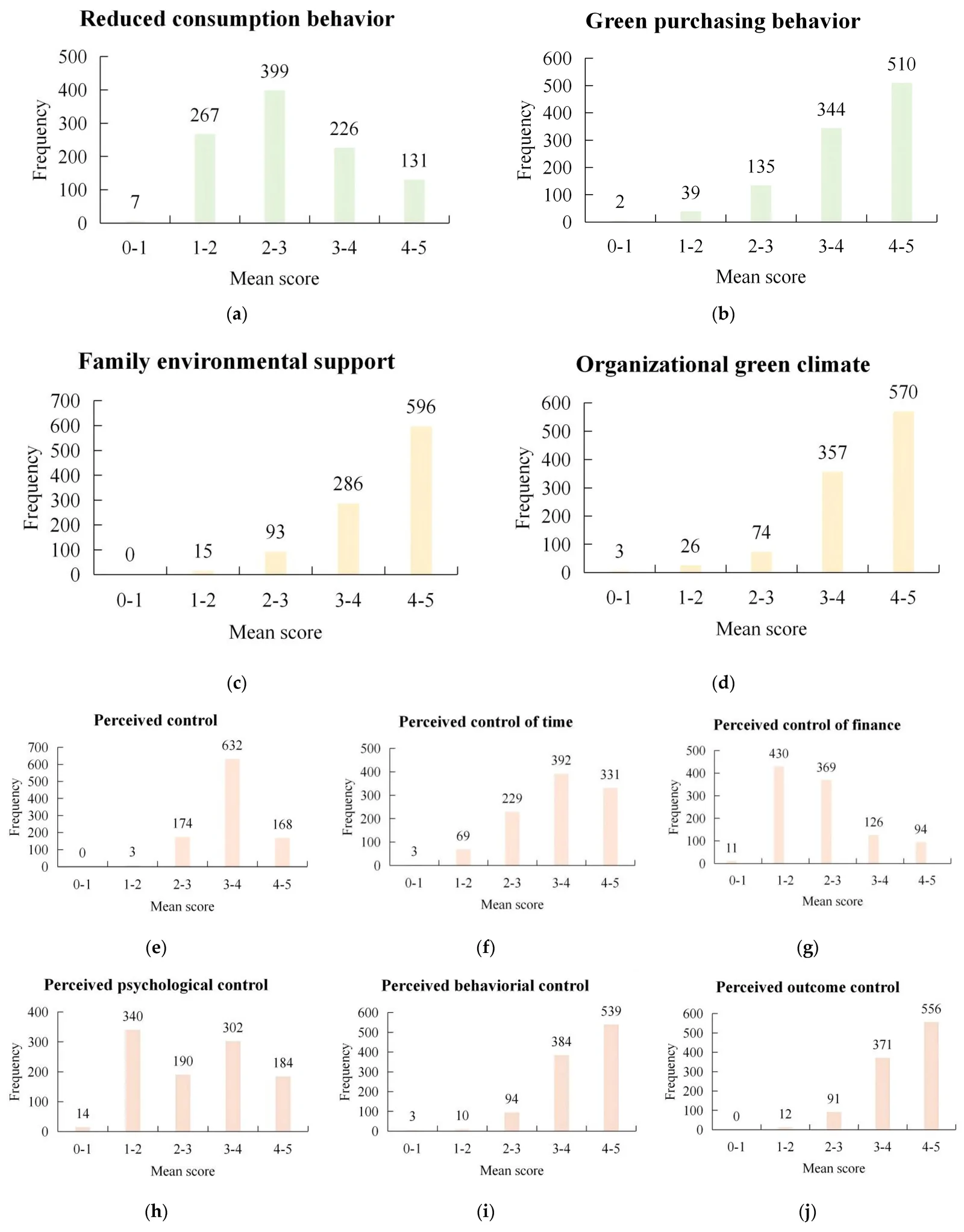
Distribution of mediating, moderating, and dependent variables. Note: (**a**) shows the score distribution of reduced consumption behavior in the sample, (**b**) presents the score distribution of green purchasing behavior, (**c**) displays the score distribution of family environmental support, (**d**) shows the score distribution of organizational green climate, (**e**) illustrates the overall score distribution of perceived control, (**f**) shows the score distribution of perceived control of time, (**g**) presents the score distribution of perceived control of finance, (**h**) illustrates the score distribution of perceived psychological control, (**i**) displays the score distribution of perceived behavioral control, and (**j**) shows the score distribution of perceived outcome control.

As shown in Figure 3, respondents differed substantially across the two dimensions of sustainable consumption. Green purchasing behavior (M = 3.90) was more prevalent than reduced consumption behavior (M = 2.85). This pattern may indicate that public communication about green consumption has encouraged employees to consider environmental attributes when selecting products. It may also reflect the greater difficulty of reduced consumption, which requires sustained self-restraint, compared with choosing greener alternatives.

The dimensions of perceived control also differed markedly. Mean scores, in descending order, were perceived outcome control (M = 4.06), perceived behavioral control (M = 4.04), perceived control of time (M = 3.55), perceived psychological control (M = 2.92), and perceived control of finances (M = 2.52). The relatively high levels of outcome and behavioral control indicate that respondents generally believed green consumption could improve environmental outcomes and that environmentally responsible consumption was feasible. Perceived control of time was moderate, consistent with some erosion of time autonomy through overtime. By contrast, the relatively low levels of psychological and financial control suggest that emotional strain and financial pressure were important constraints on respondents’ overall sense of control. Both family emotional support (M = 4.00) and organizational green climate (M = 4.01) were relatively high, indicating generally supportive family relationships and strong environmental values within respondents’ organizations.

## 4. Results

### 4.1. Reliability and Validity Test

#### 4.1.1. Internal Consistency Reliability Test

After verifying the construct validity of the key scales, Cronbach’s α coefficients were used to evaluate internal consistency, and Corrected Item-Total Correlation (CITC) was adopted to assist in examining item suitability. CITC reflects the correlation between a single item and the total score of its corresponding dimension; higher values indicate a stronger match between the item and the core connotation of the dimension. As shown in Table 2, the CITC values for all scales exceed the cutoff criterion of 0.4, suggesting satisfactory item convergence within each dimension. Meanwhile, all Cronbach’s α coefficients surpass the widely accepted threshold of 0.70, demonstrating desirable internal consistency of the scales.

#### 4.1.2. Exploratory Factor Analysis and Structural Validity

To determine whether the adapted scales for overtime motivations and perceived control were consistent with the proposed factor structures, separate exploratory factor analyses (EFAs) were conducted. The Kaiser–Meyer–Olkin (KMO) measure of sampling adequacy and Bartlett’s test of sphericity were first used to assess the suitability of the data for factor analysis.

The EFA of the overtime motivation scale yielded a KMO value of 0.825, and Bartlett’s test of sphericity was significant ($\chi^{2}$ = 7352.313, *p* < 0.001), confirming the suitability of the data for factor analysis. Principal component analysis with varimax rotation extracted six factors with eigenvalues greater than 1, which jointly explained 68.581% of the variance. All items loaded above 0.60 on their expected factors, with no substantial cross-loadings. The six factors, including development-driven, subsistence-driven, belonging-driven, escape-type, peer-pressure, and mandatory overtime, were consistent with the proposed structure. The factor-loading matrix is presented in Table 3.

The EFA of the perceived control scale yielded a KMO value of 0.776, and Bartlett’s test of sphericity was significant ($\chi^{2}$ = 5275.760, *p* < 0.001). Principal component analysis with varimax rotation extracted five factors with eigenvalues greater than 1, which jointly explained 69.158% of the variance. All items loaded satisfactorily on their intended dimensions, including time, financial, psychological, behavioral, and outcome control, with low cross-loadings. These results support the stability and validity of the adapted five-factor structure. The factor-loading matrix is presented in Table 4.

Taken together, these findings indicate that the contextually adapted overtime motivation and perceived control scales demonstrated satisfactory structural validity in this sample and that the measurement structure was consistent with the theoretical model.

#### 4.1.3. Confirmatory Factor Analysis

**Figure 4:**
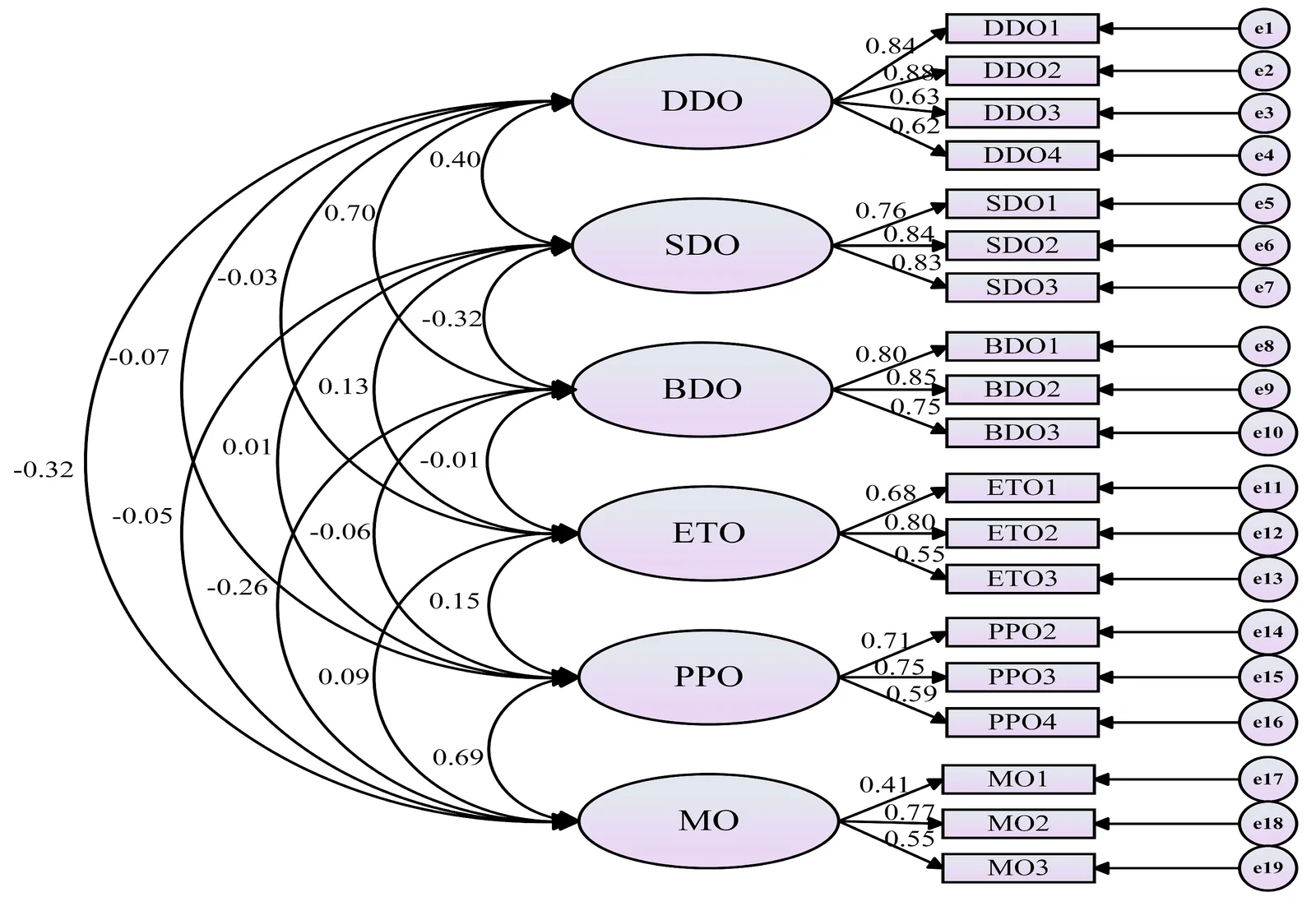
Confirmatory factor analysis model path diagram.

To further test the model fit, convergent validity and discriminant validity of the measurement model for the six types of overtime motivations, this study constructed a confirmatory factor analysis model with six latent overtime variables. The path structure of the model is illustrated in Figure 4. The overall model fit results are as follows: $\chi^{2}$/df = 3.907 < 5; RMSEA = 0.053 < 0.08; GFI = 0.949 > 0.9; NFI = 0.928 > 0.9; TLI = 0.931 > 0.9; CFI = 0.945 > 0.9. Taken together, all core fit indices indicate that the measurement model of the six overtime motivations achieves satisfactory overall fit.

The results for convergent validity show that the composite reliability (CR) of the six latent overtime variables ranges from 0.71 to 0.89 (all exceeding the cutoff of 0.7), and the average variance extracted (AVE) of each dimension ranges from 0.52 to 0.70 (all above the 0.5 threshold). The standardized factor loading of all items exceeds 0.6, confirming acceptable convergent validity. For discriminant validity, the square root of the AVE for each overtime dimension is greater than the Pearson correlation coefficient between that construct and all other overtime dimensions. Combined with the correlation matrix, this evidence supports adequate discriminant validity across the six overtime motivation constructs and rules out severe construct overlap.

#### 4.1.4. Common Method Bias and Multicollinearity Tests

Because all variables were measured using employee self-reports, Harman’s single-factor test was conducted to assess common method bias (Podsakoff et al., 2003). An unrotated exploratory factor analysis of all core measurement items extracted 14 factors with eigenvalues greater than 1. The first factor accounted for 19.728% of the variance, well below the conventional threshold of 40%, and no single factor explained most of the variance. These results suggest that common method bias was unlikely to seriously affect the subsequent analyses. The results are presented in Table 5.

Harman’s single-factor test was further conducted for the six-dimensional overtime motivation scale. An unrotated exploratory factor analysis was performed on all overtime-motivation items. Six common factors with eigenvalues greater than 1 were extracted, and the first common factor accounted for 24.737% of the variance, which is below the 40% cutoff. This suggests that the risk of common method bias within the overtime motivation measures is acceptable.

The Pearson correlation matrix for the six overtime motivation variables is presented in Table 6. The maximum correlation coefficient among these variables is 0.609, which does not reach the 0.7 threshold for high correlation. A subsequent multicollinearity test yields variance inflation factor (VIF) values ranging from 1.029 to 1.743; all values fall within an acceptable range. The results indicate no serious multicollinearity among the six overtime motivation constructs, justifying their inclusion in subsequent model analyses.

### 4.2. Regression Analysis

Separate models were estimated for reduced consumption behavior and green purchasing behavior. Models 1 and 6 included only the control variables and served as baselines. Models 2 and 7 added the six overtime motivations to test their associations with the outcome variables. Model 5 examined the associations between overtime motivations and perceived control. Models 3 and 8 estimated the association between perceived control and each outcome. Finally, Models 4 and 9 included the control variables, overtime motivations, and perceived control simultaneously. Changes in the coefficients for overtime motivations between Models 2 and 4 and between Models 7 and 9 were used to assess mediation.

#### 4.2.1. Regression Results for Reduced Consumption Behavior

As shown in Table 7, Model 1 included only the control variables and explained 2.4% of the variance in reduced consumption behavior (R^2^ = 0.024). Adding the six overtime motivations in Model 2 significantly increased explained variance (ΔR^2^ = 0.074, *p* < 0.01). Specifically, escape-type overtime (β = −0.117, *p* < 0.01), peer-pressure overtime (β = −0.084, *p* < 0.05), and mandatory overtime (β = −0.172, *p* < 0.001) were negatively associated with reduced consumption behavior. H1b was partially supported.

#### 4.2.2. Regression Results for Green Purchasing Behavior

As shown in Table 7, Model 6 included only the control variables and explained 5.1% of the variance. Adding the overtime motivations in Model 7 increased explained variance substantially (ΔR^2^ = 0.330). Development-driven overtime (β = 0.106, *p* < 0.001), subsistence-driven overtime (β = 0.067, *p* < 0.001), and belonging-driven overtime (β = 0.358, *p* < 0.001) were positively associated with green purchasing behavior, whereas the reactive overtime motivations were not significant. H1a was partially supported.

#### 4.2.3. Mediation Regression Results for Perceived Control

As shown in Table 7, Model 5 takes perceived control as the dependent variable to examine the associations between the six overtime motivations and overall perceived control. The results show that development-driven overtime (β = 0.080, *p* < 0.001), subsistence-driven overtime (β = 0.039, *p* < 0.001), and belonging-driven overtime (β = 0.231, *p* < 0.001) are significantly and positively associated with perceived control; escape-type overtime (β = −0.061, *p* < 0.001), peer-pressure-driven overtime (β = −0.060, *p* < 0.001), and mandatory overtime (β = −0.044, *p* < 0.01) are significantly and negatively associated with perceived control, satisfying the prerequisite for mediation testing.

Regarding reduced consumption behavior (Models 3 and 4), perceived control is insignificant in Model 3 and remains statistically nonsignificant in Model 4, which includes control variables, overtime motivations, and perceived control. This model explains 7.6% of the variance. These findings indicate that perceived control does not mediate the association between reactive overtime motivations and reduced consumption behavior.

For green purchasing behavior (Models 8 and 9), perceived control is positively associated with green purchasing behavior in Model 8 (β = 0.669, *p* < 0.001). Model 9 incorporates control variables and overtime motivations and raises the explained variance to 0.354; perceived control remains statistically significant. Meanwhile, the coefficients of development-driven, subsistence-driven, and belonging-driven overtime decline from 0.106 to 0.081, 0.067 to 0.054, and 0.358 to 0.287, respectively. This pattern is consistent with partial mediation.

### 4.3. Mediation Analysis

To examine the specific dimensions through which perceived control mediates the relationships between overtime motivations and sustainable consumption behavior, bias-corrected bootstrap analyses with 5000 resamples were conducted. The five mediators were perceived control of time (PCT), perceived control of finances (PCF), perceived psychological control (PPC), perceived behavioral control (PBC), and perceived outcome control (POC).

As shown in Table 8, several dimensions of perceived control partially mediated the relationships between proactive overtime motivations and green purchasing behavior. Development-driven overtime (DDO) had significant indirect effects through perceived control of time, finances, behavior, and outcomes, with effect sizes of 0.0623, 0.0276, 0.0899, and 0.1605, respectively; the direct effects remained significant. Subsistence-driven overtime (SDO) had significant indirect effects through perceived control of time, finances, behavior, and outcomes. Belonging-driven overtime (BDO) likewise had significant indirect effects through perceived control of time, behavior, and outcomes. By contrast, none of the perceived control dimensions significantly mediated the relationships between reactive overtime motivations and reduced consumption behavior, consistent with the hierarchical regression results. Thus, H2a was supported, whereas H2b was not supported.

### 4.4. Moderation Analysis

Moderation analyses were conducted using PROCESS Model 1 in SPSS 27, with 5000 bootstrap resamples. The independent variables, moderators, and interaction terms were mean-centered. Because of space constraints, only statistically significant moderation results are reported.

As shown in Table 9, family emotional support moderated the negative relationship between peer-pressure overtime and reduced consumption behavior (β = −0.1006, *p* < 0.05) and the negative relationship between mandatory overtime and reduced consumption behavior (β = −0.1251, *p* < 0.01). Organizational green climate also strengthened the positive relationship between belonging-driven overtime and green purchasing behavior (β = 0.0641, *p* < 0.05). No other interaction term was significant. Thus, H3 and H4 were partially supported.

**Figure 5:**
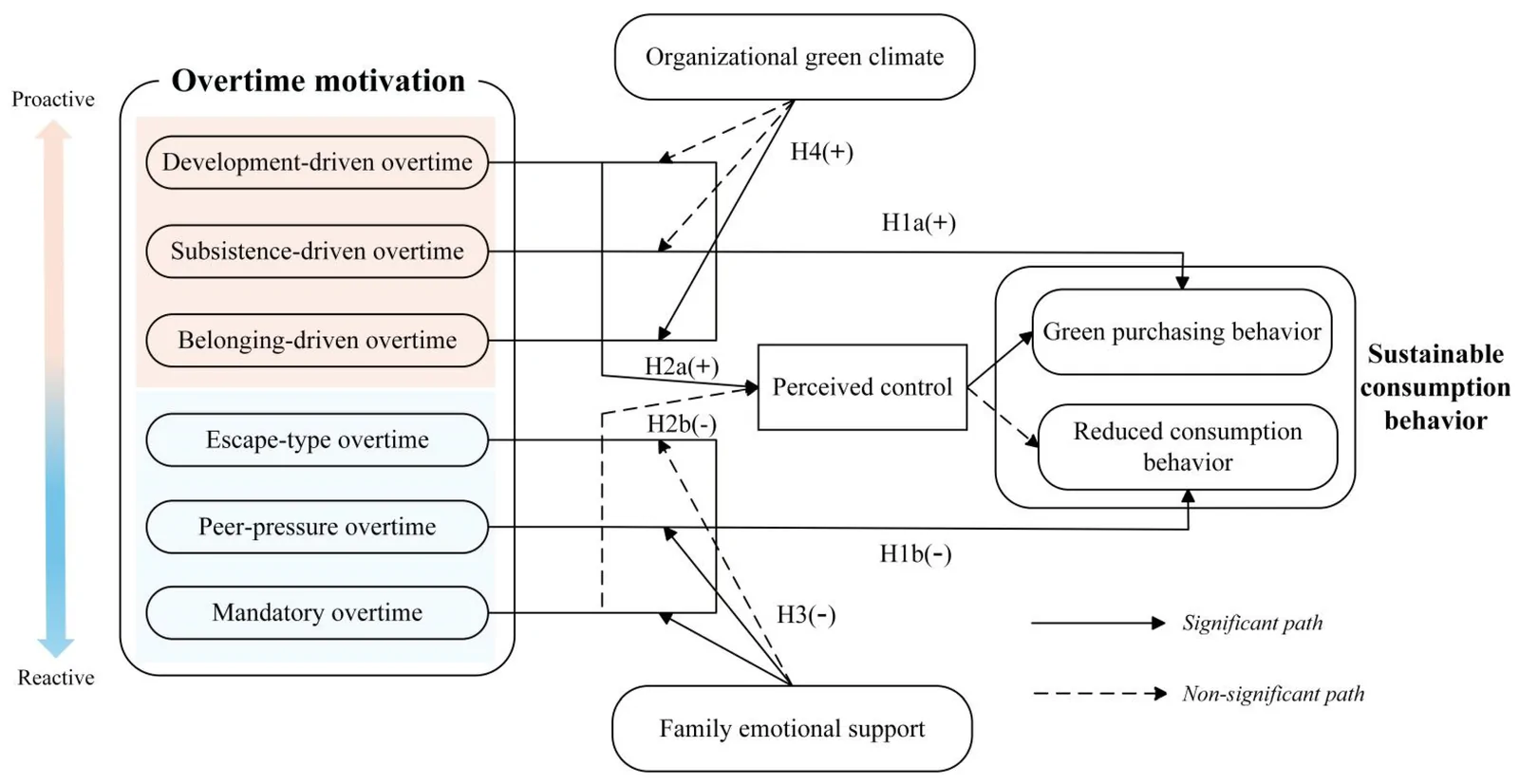
Hypothesis testing diagram.

The results of all hypothesis tests in this study are summarized in Figure 5.

## 5. Discussion

### 5.1. Differential Associations of Overtime Motivations with Sustainable Consumption Behavior

The results reveal a differentiated pattern in which proactive overtime motivations were positively associated with green purchasing behavior, whereas reactive overtime motivations were negatively associated with reduced consumption behavior. This pattern indicates that overtime should not be treated simply as a uniform job demand or as a matter of working hours alone. Rather, the motivational meaning individuals attribute to overtime work is associated with whether overtime is experienced as resource gain or resource depletion. From the perspectives of conservation of resources theory and ego depletion theory, proactive overtime may provide psychological, social, or material resources that support value-consistent consumption, whereas reactive overtime may drain the self-regulatory resources needed to restrain consumption (S. Kim & Rucker, 2012). The distinction between green purchasing and reduced consumption is therefore important: green purchasing may be facilitated by resource availability and positive value expression, while reduced consumption is especially vulnerable to depletion because it requires inhibition and restraint.

The positive associations between development-driven, subsistence-driven, and belonging-driven overtime and green purchasing suggest that proactively motivated overtime can spill over into private consumption through resource accumulation and meaning transfer (Dong et al., 2025). Development-driven overtime may strengthen competence and efficacy through challenging work; subsistence-driven overtime may improve perceived financial security and reduce sensitivity to green price premiums; and belonging-driven overtime may reinforce organizational identification and value consistency. These resources may be linked to employees’ willingness and ability to choose environmentally friendly products. The three proactive overtime motivations exhibit no significant association with reduced consumption, potentially because the two forms of sustainable consumption place different demands on psychological resources. Green purchasing involves deliberate product screening, whereby sufficient cognitive resources alone support comparisons of environmental attributes (Zhang et al., 2024). Reduced consumption, by contrast, requires enduring self-restraint and continuously drains self-control resources (Haynes et al., 2016). The positive resources accrued from proactive overtime can facilitate eco-friendly purchasing but are inadequate to sustain the self-regulation required for long-term consumption restraint (J. C. Kim et al., 2019). Importantly, this interpretation does not imply that longer working hours are inherently beneficial. Rather, it suggests that when overtime is experienced as self-endorsed, meaningful, or materially supportive, its psychological consequences may differ from those of externally imposed overtime (Cooper & Lu, 2019).

The negative associations between reactive overtime motivations and reduced consumption are consistent with differentiated explanatory pathways. Escape-type, peer-pressure, and mandatory overtime are likely to be experienced as constraints on autonomy and time, thereby intensifying fatigue and reducing the capacity for self-regulation (Park et al., 2024). Under such conditions, consumption may shift from long-term planning toward short-term affect regulation or symbolic compensation. This helps explain why reactive overtime was more clearly related to reduced consumption than to green purchasing: restraint-oriented behavior may be more difficult to sustain when employees lack the psychological energy needed to resist unnecessary consumption. These findings extend research on the cross-domain influence of workplace experiences on individuals’ everyday sustainable consumption behaviors by showing that work pressure may influence not only well-being and family life but also everyday consumer decisions with environmental consequences.

### 5.2. The Differential Mediating Role of Perceived Control

The mediation results provide evidence on the indirect associations linking overtime motivation to sustainable consumption. Perceived control showed significant indirect associations between proactive overtime motivations and green purchasing behavior, but it did not mediate the relationships between reactive overtime motivations and reduced consumption behavior. This asymmetry suggests that perceived control operates primarily as a deliberative pathway rather than as a universal mechanism explaining all forms of sustainable consumption.

For proactive overtime, perceived control appears to translate work-derived resource gains into environmentally oriented purchasing choices. From a resource caravan perspective of conservation of resources theory, competence, financial security, organizational identification, and perceived control should not be viewed as isolated resources. Instead, they may form an interconnected set of personal, material, and social resources that enables employees to make value-consistent consumption decisions (Hobfoll, 2011). Development-driven overtime may contribute to competence and efficacy; subsistence-driven overtime may enhance financial security; and belonging-driven overtime may strengthen social identification and value alignment. These resource gains can reinforce employees’ perceived control over time, finances, behavior, and outcomes, thereby increasing their confidence and capacity to search for, evaluate, and select green alternatives (Cooper & Lu, 2019).

Green purchasing may, in turn, help sustain this resource caravan. It may generate or reinforce valued resources, including environmental self-efficacy, moral satisfaction, identity consistency, and perceived alignment with organizational or societal environmental values. Thus, proactively motivated overtime contributes to a broader resource caravan that both enables and is potentially reinforced by green purchasing behavior. This finding is consistent with pro-environmental behavior research showing that perceived behavioral control is a key antecedent of environmental action (Bamberg & Möser, 2007), and it extends that literature by identifying work-related motivational experiences as a potential source of resource-caravan enrichment.

By contrast, the absence of mediation for reactive overtime suggests that its association with reduced consumption may not involve a reflective sense of control. Drawing on the conservation of resources theory (Hobfoll, 1989), reactive overtime may be perceived by individuals as a resource threat and may be associated with multidimensional resource depletion. Originating from external mandates rather than employees’ voluntary choices, such overtime unexpectedly encroaches on discretionary personal time, constraining autonomy over leisure, financial planning and consumption decisions. It continuously drains core psychological resources such as energy and emotion and undermines employees’ perceived autonomy over daily life affairs. Consistent with the empirical findings of this study, reactive overtime is negatively associated with reduced consumption behavior, but the estimated indirect association through perceived control is not statistically significant. Constrained by the exclusion of observed variables such as emotional exhaustion, impulsivity, and compensatory consumption motives, the current empirical model cannot fully unpack the potential processes linking reactive overtime and consumption reduction. Prior literature suggests that sustained resource depletion predisposes individuals toward short-term affect-driven consumption coping behaviors (Hagger et al., 2010). Under such circumstances, employees’ consumption behaviors may serve as an immediate coping mechanism following resource loss. This interpretation helps explain why perceived control was not a significant mediating mechanism for reduced consumption and highlights the need for future research to examine emotional exhaustion, compensatory consumption motives, and impulsivity as alternative mechanisms.

### 5.3. Differentiated Moderating Roles of Family Emotional Support and Organizational Green Climate

The moderation findings also qualify the assumed protective role of social context. Family emotional support intensified, rather than buffered, the negative relationships between peer-pressure and mandatory overtime and reduced consumption. It suggests that family support may operate as a value-amplifying context rather than merely as a buffering resource. Employees who experience greater warmth and emotional fulfillment in family life may regard nonwork time as more valuable. Consequently, when externally imposed overtime deprives them of opportunities to participate in family life, they may experience stronger frustration, guilt, or loss of autonomy (Aarntzen et al., 2019). Compensatory consumption may then become a way to restore emotional balance or symbolically compensate for the loss of valued family time. This result should not be interpreted as evidence that family support is generally harmful. Instead, it suggests that supportive family relationships may create stronger expectations for presence, reciprocity, and care.

Organizational green climate showed a more selective moderating effect: it strengthened the positive association between belonging-driven overtime and green purchasing behavior. This pattern is consistent with Zafar et al. (2023). Employees who work overtime out of organizational belonging are likely to be more receptive to organizational values; when the organization strongly communicates environmental norms, these employees may internalize those values and carry them into private consumption. The finding extends research on organizational green climate by suggesting that its association with green purchasing may depend on employees’ motivational attachment to the organization. In other words, green climate may be most likely to spill over beyond the workplace when employees already perceive the organization as part of their social identity.

Taken together, these findings should be understood within the cultural and organizational context in which overtime occurs. In the Chinese workplace, overtime may convey economic necessity, career commitment, relational obligation, or organizational loyalty, and these meanings may shape whether overtime is experienced as self-endorsed engagement or external pressure (Dong et al., 2025). By distinguishing among overtime motivations, this study provides a more context-sensitive account of how work experiences enter the consumption domain. At the same time, the framework has broader relevance for work-consumption research because it suggests that the sustainability consequences of work depend not only on workload but also on the motivational and social meanings attached to work.

### 5.4. Theoretical Implications and Limitations

This study offers several theoretical implications. First, it extends research on overtime by shifting attention from the amount of time worked to the motivational meaning of overtime. The findings suggest that overtime should not be treated as a uniformly harmful job demand; rather, its associations with sustainable consumption differ according to whether employees experience it as self-endorsed, resource-generating, socially pressured, or externally imposed. Second, by revealing that sustainable consumption behaviors may follow distinct psychological pathways, this study further refines the analytical logic of work–life spillover behaviors. Proactive overtime motivations were linked to green purchasing partly through perceived control, whereas reactive overtime motivations were associated with reduced consumption without operating through perceived control. This pattern suggests that work-to-consumption spillover may involve both deliberative control-based processes and more affective or compensatory processes. Third, the study advances sustainable consumption research by showing that green purchasing and reduced consumption are differently associated with work-related experiences. Treating sustainable consumption as a multidimensional construct helps explain why workplace experiences may be positively associated with environmentally preferable purchasing while being negatively associated with consumption restraint.

Several limitations should be acknowledged. First, this study adopted a cross-sectional, one-shot questionnaire to collect data, which can only confirm correlational associations rather than precisely establish the dynamic causal ordering of variables. Constructs such as overtime motivation and perceived control fluctuate within days. The Experience Sampling Method (ESM) captures momentary shifts across multiple time points and can better clarify temporal ordering. Nevertheless, this design was not implemented here due to practical constraints. Future work may deploy ESM with multi-day tracking to dynamically test the processes linking these variables. Second, all variables were measured through self-reports, which may introduce common method bias despite the diagnostic tests reported above. Future research could incorporate behavioral consumption data, supervisor reports, or household-level observations. Third, the sample consisted of Chinese employees, and the findings may reflect local overtime norms, labor-market pressures, and family expectations. Comparative studies across cultural and institutional contexts would help assess the generalizability of the proposed framework. Finally, future research could examine additional mechanisms, such as emotional exhaustion, guilt, impulsivity, and compensatory consumption, to explain why reactive overtime affects reduced consumption outside the perceived-control pathway.

## 6. Conclusions and Implications

### 6.1. Conclusions

Based on the survey data of 1030 employees, this study systematically explores the differential associations of overtime motivations with employees’ sustainable consumption behavior and assesses the indirect associations through perceived control and the moderating roles of family emotional support and organizational green climate. The main conclusions are as follows.

(1) Overtime motivations were differentially associated with sustainable consumption behavior. Proactive motivations were positively associated with green purchasing behavior, with the largest association observed for belonging-driven overtime, followed by development-driven and subsistence-driven overtime. Reactive motivations were negatively associated with reduced consumption behavior, with the largest association observed for mandatory overtime, followed by escape-type and peer-pressure overtime.

(2) Perceived control partially mediated the relationships between proactive overtime motivations (development-driven, subsistence-driven, and belonging-driven) and green purchasing behavior but did not mediate the relationships between reactive overtime motivations and reduced consumption behavior. Development-driven overtime had indirect effects through perceived control of time, finances, behavior, and outcomes. Subsistence-driven overtime had indirect effects through perceived control of time, behavior, and outcomes. Belonging-driven overtime had indirect effects through perceived control of time, behavior, and outcomes.

(3) Family emotional support significantly strengthened the negative associations of peer-pressure overtime and mandatory overtime with employees’ reduced consumption behavior. The higher the level of family emotional support, the stronger the negative associations of these two types of reactive overtime with employees’ reduced consumption. Meanwhile, organizational green climate moderated the positive association between belonging-driven overtime and employees’ green purchasing behavior. The stronger the organizational green climate, the stronger the positive association between belonging-driven overtime and employees’ green purchasing behavior.

### 6.2. Policy Recommendations

**Figure 6:**
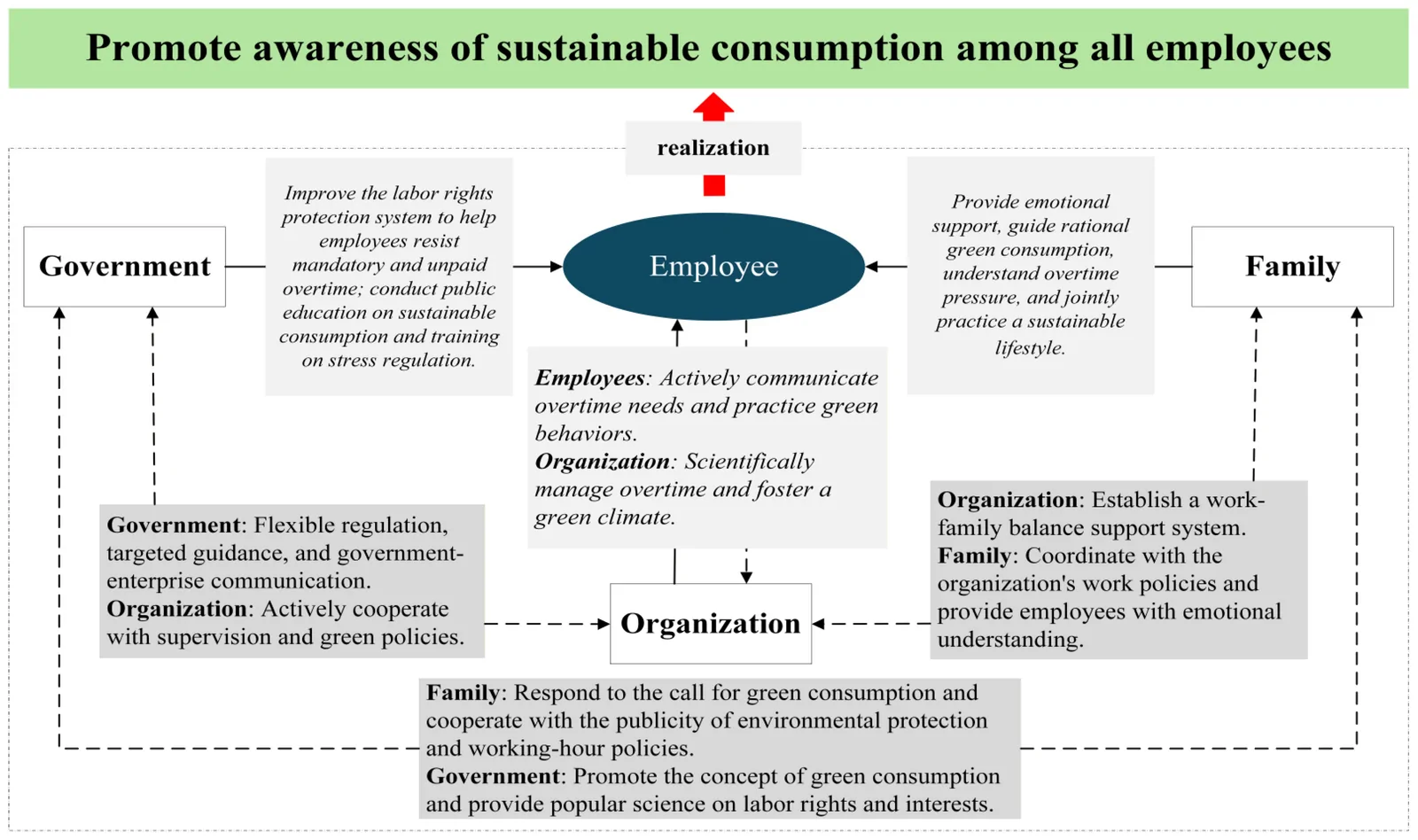
Policy recommendations.

Based on the above findings, recommendations are proposed for four groups of stakeholders, as summarized in Figure 6.

(1) Employees should proactively identify their own overtime motivation and distinguish between changes in psychological resources brought by active and passive overtime. They should alleviate emotional exhaustion from passive overtime through non-consumptive methods such as exercise and meditation, and develop rational consumption habits. By adopting shopping cooling-off periods and green consumption lists, they can translate environmental concepts into daily consumption behaviors, balancing personal psychological state and sustainable consumption responsibilities.

(2) Families should provide inclusive emotional support for overtime employees, preventing excessive consumption driven by guilt caused by overtime. Meanwhile, families should cultivate a green and rational consumption atmosphere, integrate reduced consumption and green purchasing into daily family life, and form consumption behavior coordination with employees. Positive guidance from the family context may help mitigate the negative association between passive overtime and sustainable consumption.

(3) Enterprises should optimize working hour management to reduce passive overtime such as mandatory and unpaid overtime. They should guide employees to form active and positive work engagement through reasonable compensation and career development. At the same time, enterprises should build a strong green organizational climate, integrate environmental concepts into management and culture, and encourage employees to extend the organization’s environmental values to personal consumption, realizing the unity of green behavior in work and life domains.

(4) The government should improve the labor rights and interest protection system, strengthen supervision over enterprises’ excessive overtime and invisible overtime, and safeguard employees’ right to rest and time autonomy. Meanwhile, the government should carry out public publicity of sustainable consumption and psychological counseling services, incentivize enterprises to fulfill employee well-being and ecological responsibilities through taxation and green procurement policies, and build a social environment where work, consumption and ecology interact positively.

## Figures and Tables

**Table 1:** Sample characteristics.

Variable		Frequency	Proportion
Gender	Male	470	45.6%
	Female	560	54.4%
Age	≤24	68	6.60%
	25–29	75	7.28%
	30–34	188	18.25%
	35–39	377	36.60%
	40–44	233	22.62%
	≥45	89	8.64%
Education background	High school/technical school	28	2.72%
	Junior college	129	12.52%
	Bachelor’s degree	808	78.45%
	Graduate degree	65	6.31%
Monthly household income	¥2001–¥5000	103	10.00%
	¥5001–¥10,000	493	47.86%
	¥10,001–¥20,000	373	36.21%
	≥¥20,000	61	5.92%
Type of occupation	Staff of State organs, party organizations, institutions and state enterprises	176	17.09%
	Professional and technical staff	347	33.69%
	Clerical and related personnel	258	25.05%
	Commercial, service workers	152	14.76%
	Production and transport equipment operators and related personnel	84	8.16%
	Other	13	1.26%
Nature of the organization	Government department	22	2.14%
	Public institution	93	9.03%
	State-owned enterprise	175	16.99%
	Collective-owned enterprise	20	1.94%
	Joint-stock enterprise	86	8.35%
	Foreign-funded enterprise	56	5.44%
	Chinese-foreign equity joint venture	32	3.11%
	Chinese-foreign cooperative joint venture	6	0.58%
	Private enterprise	539	52.33%
	Other	1	0.10%
Size of the organization	≤55	115	11.17%
	56–100	225	21.84%
	101–500	442	42.91%
	≥501	248	24.08%

**Table 2:** Reliability analysis results.

Variables		Abbreviation	*n*	Cronbach’s α	CITC
Overtime motivation	Development-driven overtime	DDO	4	0.829	0.573–0.763
	Subsistence-driven overtime	SDO	3	0.852	0.684–0.751
	Belonging-driven overtime	BDO	3	0.839	0.680–0.731
	Escape-type overtime	ETO	3	0.703	0.472–0.596
	Peer-pressure overtime	PPO	3	0.722	0.481–0.590
	Mandatory overtime	MO	3	0.712	0.500–0.566
Perceived control	Perceived control of time	PCT	3	0.775	0.567–0.686
	Perceived control of finance	PCF	3	0.786	0.482–0.709
	Perceived psychological control	PPC	3	0.871	0.731–0.781
	Perceived behavioral control	PBC	3	0.708	0.486–0.567
	Perceived outcome control	POC	3	0.701	0.479–0.558
Family emotional support		FES	10	0.887	0.532–0.697
Organizational green climate		OGC	4	0.830	0.627–0.676
Green consumption behavior	Reduced consumption behavior	RCB	3	0.775	0.485–0.710
	Green purchasing behavior	GPB	3	0.808	0.652–0.671

**Table 3:** Principal component analysis results of the overtime motivation scale.

Indicator	Components					
	1	2	3	4	5	6
DDO1	0.760	0.182	0.310	−0.049	−0.006	−0.097
DDO2	0.808	0.175	0.270	−0.047	−0.001	−0.149
DDO3	0.711	−0.072	0.320	−0.062	0.043	−0.041
DDO4	0.742	0.280	0.089	0.085	−0.008	0.026
SDO1	0.247	0.802	0.147	0.057	0.036	−0.030
SDO2	0.080	0.881	0.086	−0.033	0.033	0.033
SDO3	0.125	0.852	0.077	0.002	0.116	0.030
BDO1	0.244	0.103	0.845	0.002	−0.003	−0.038
BDO2	0.342	0.139	0.795	0.003	0.003	−0.058
BDO3	0.261	0.096	0.797	−0.075	−0.017	−0.048
ETO1	0.033	0.098	−0.045	0.096	0.786	−0.003
ETO2	0.049	−0.017	−0.038	0.039	0.849	0.004
ETO3	−0.057	0.070	0.066	−0.001	0.744	0.045
PPO2	−0.008	0.020	0.029	0.777	0.041	0.148
PPO3	0.049	−0.006	−0.074	0.811	0.046	0.118
PPO4	−0.056	0.015	0.006	0.756	0.044	0.070
MO1	−0.038	0.210	−0.011	0.025	−0.024	0.814
MO2	−0.083	−0.018	−0.152	0.469	0.010	0.601
MO3	−0.119	−0.228	−0.039	0.289	0.092	0.627

**Table 4:** Principal component analysis results of the perceived control scale.

Indicator	Components				
	1	2	3	4	5
PCT1	−0.063	0.783	0.042	0.079	0.145
PCT2	−0.129	0.786	0.088	0.174	0.102
PCT3	−0.103	0.844	0.108	0.098	0.141
PCF1	0.039	0.049	0.896	0.049	−0.004
PCF2	0.045	0.027	0.901	0.027	0.023
PCF3	−0.126	0.208	0.644	0.028	0.303
PPC1	0.898	−0.093	0.021	−0.046	−0.035
PPC2	0.883	−0.143	−0.039	−0.063	0.001
PPC3	0.876	−0.056	0.007	−0.038	−0.081
PBC1	−0.018	0.211	0.184	0.331	0.566
PBC2	−0.047	0.104	0.118	0.159	0.785
PBC3	−0.039	0.125	−0.014	0.061	0.792
POC1	−0.067	0.145	0.096	0.829	0.015
POC2	−0.028	0.109	−0.011	0.769	0.129
POC3	−0.052	0.065	0.001	0.634	0.329

**Table 5:** Common method bias test.

Factor	Eigenvalue	Variance Explained (%)	Cumulative Variance Explained (%)
1	11.442	19.728	19.728
2	3.638	6.272	26.001
3	3.229	5.567	31.568
4	2.666	4.597	36.164
5	2.454	4.231	40.396
6	2.042	3.521	43.917
7	1.823	3.142	47.059
8	1.734	2.989	50.048
9	1.537	2.649	52.698
10	1.397	2.409	55.107
11	1.282	2.21	57.317
12	1.179	2.033	59.35
13	1.132	1.952	61.302
14	1.045	1.802	63.104

**Table 6:** Correlation matrix of overtime motivations.

Variables	SAM	EAM	EPO	EWM	OA	MO	VIF
SAM	1	-	-	-	-	-	1.743
EAM	0.366 ***	1	-	-	-	-	1.186
EPO	0.609 ***	0.285 ***	1	-	-	-	1.610
EWM	0.022	0.123 ***	0.000	1	-	-	1.029
OA	−0.038	0.015	−0.057	0.111 ***	1	-	1.285
MO	−0.215 ***	−0.021	−0.182 ***	0.066 *	0.460 ***	1	1.344

Note: * *p* < 0.05; *** *p* < 0.001.

**Table 7:** Hierarchical regression analysis results.

Variables	Reduced Consumption Behavior				PC	Green Purchasing Behavior			
	Model 1	Model 2	Model 3	Model 4	Model 5	Model 6	Model 7	Model 8	Model 9
DDO		−0.047 (0.039)		−0.038 (0.040)	0.080 *** (0.017)		0.106 *** (0.027)		0.081 ** (0.027)
SDO		0.013 (0.028)		0.018 (0.028)	0.039 *** (0.012)		0.067 *** (0.019)		0.054 ** (0.019)
BDO		0.008 (0.042)		0.035 (0.045)	0.231 *** (0.018)		0.358 *** (0.028)		0.287 *** (0.030)
ETO		−0.117 ** (0.036)		−0.124 *** (0.036)	−0.061 *** (0.015)		−0.039 (0.025)		−0.020 (0.024)
PPO		−0.084 * (0.034)		−0.091 ** (0.034)	−0.060 *** (0.014)		0.021 (0.023)		0.040 (0.023)
MO		−0.172 *** (0.038)		−0.177 *** (0.038)	−0.044 ** (0.016)		0.006 (0.026)		0.020 (0.026)
PC			0.022 (0.060)	−0.116 (0.074)				0.669 *** (0.043)	0.308 *** (0.050)
Age	−0.103 *** (0.023)	−0.104 *** (0.024)	−0.103 *** (0.024)	−0.102 *** (0.024)	0.017 (0.010)	−0.048 * (0.019)	−0.041 ** (0.016)	−0.056 *** (0.017)	−0.046 ** (0.016)
Sex	−0.116 * (0.059)	−0.140 * (0.058)	−0.116 * (0.059)	−0.138 * (0.058)	0.020 (0.024)	0.039 (0.047)	0.077 (0.040)	0.030 (0.042)	0.071 (0.039)
Education level	0.049 (0.060)	0.076 (0.060)	0.033 (0.046)	0.077 (0.060)	0.008 (0.025)	−0.137 ** (0.048)	−0.040 (0.041)	−0.093 * (0.043)	−0.042 (0.040)
Income	0.037 (0.044)	−0.000 (0.044)	0.022 (0.060)	0.012 (0.045)	0.104 *** (0.019)	0.226 *** (0.035)	0.081 ** (0.030)	0.083 ** (0.033)	0.049 (0.030)
Constant	3.112 *** (0.246)	4.364 *** (0.319)	3.048 *** (0.303)	4.626 *** (0.359)	2.259 *** (0.135)	3.791 *** (0.195)	2.010 *** (0.218)	1.843 *** (0.216)	1.315 *** (0.242)
F	7.324 ***	9.247 ***	5.880 ***	8.643 ***	74.476 ***	14.756 ***	51.681 ***	62.844 ***	52.197 ***
R^2^	0.028	0.083	0.028	0.085	0.422	0.054	0.337	0.235	0.361
ΔR^2^	0.024	0.074	0.023	0.076	0.417	0.051	0.330	0.231	0.354

Note: * *p* < 0.05; ** *p* < 0.01; *** *p* < 0.001. DDO = development-driven overtime; SDO = subsistence-driven overtime; BDO = belonging-driven overtime; ETO = escape-type overtime; PPO = peer-pressure overtime; MO = mandatory overtime; PC = perceived control.

**Table 8:** Mediation analysis results.

Path	Direct Effect		Indirect Effect	Bootstrap 95%CI		Mediating Effect
	Estimate	*p*	Estimate	Lower 2.5%	Upper 97.5%	
DDO→PCT→GPB	0.2947 ***	0.0000	0.0623	0.0363	0.0889	Partial mediation
DDO→PCF→GPB	0.3222 ***	0.0000	0.0276	0.0103	0.0457	Partial mediation
DDO→PBC→GPB	0.2729 ***	0.0000	0.0899	0.0652	0.1179	Partial mediation
DDO→POC→GPB	0.2169 ***	0.0000	0.1605	0.1305	0.1918	Partial mediation
SDO→PCT→GPB	0.1293 ***	0.0000	0.0580	0.0384	0.0792	Partial mediation
SDO→PCF→GPB	0.1547 ***	0.0000	0.0196	0.0076	0.0334	Partial mediation
SDO→PBC→GPB	0.1289 ***	0.0000	0.0586	0.0328	0.0849	Partial mediation
SDO→POC→GPB	0.1131 ***	0.0000	0.0825	0.0490	0.1174	Partial mediation
BDO→PCT→GPB	0.4402 ***	0.0000	0.0338	0.0054	0.0625	Partial mediation
BDO→PBC→GPB	0.3946 ***	0.0000	0.0870	0.0607	0.1163	Partial mediation
BDO→POC→GPB	0.3262 ***	0.0000	0.1668	0.1314	0.2039	Partial mediation

Note: *** *p* < 0.001. GPB = green purchasing behavior; DDO = development-driven overtime; SDO = subsistence-driven overtime; BDO = belonging-driven overtime; PCT = perceived control of time; PCF = perceived control of finance; PBC = perceived behavioral control; POC = perceived outcome control.

**Table 9:** Moderation analysis results.

Variables	(1)	Variables	(2)	Variables	(3)
	RCB		RCB		GPB
PPO	−0.1468 *** (0.0307)	MO	−0.1920 *** (0.0339)	BDO	0.2239 *** (0.0240)
		
FES	0.1507 *** (0.0431)	FES	0.1347 *** (0.0432)	OGC	0.5801 *** (0.0260)
		
PPO × FES	−0.1006 * (0.0446)	MO × FES	−0.1251 ** (0.0483)	BDO × OGC	0.0641 * (0.0257)
		

Note: * *p* < 0.05; ** *p* < 0.01; *** *p* < 0.001. RCB = reduced consumption behavior; GPB = green purchasing behavior; BDO = belonging-driven overtime; PPO = peer-pressure overtime; MO = mandatory overtime; FES = family emotional support; OGC = organizational green climate.

## Data Availability

The data presented in this study are available on request from the corresponding author. The data are not publicly available due to privacy and ethical restrictions.

## Supplementary Materials

The following supporting information can be downloaded at: https://www.mdpi.com/article/10.3390/bs16091521/s1, Table S1: Measurements.
