# Supplementary material for: Not All Overtime Is Equal: Differential Effects of Overtime Motivations on Employees’ Sustainable Consumption Behavior
Source: Behav Sci (Basel). 2026 Aug 28;16(9):1521. doi: 10.3390/bs16091521 (PMC13603599; doi:10.3390/bs16091521)
Supplement: Supplementary file 1 [file behavsci-16-01521-s001.zip › behavsci-4431546-supplementary.pdf]

Supplementary Materials

Table S1. Measurements.

Variables	Abbreviation	Definition and measurement	M	SD
Explained variables				
Reduced consumption behavior	RCB	Q1-1: It's only natural to treat myself to a little extra spending.<br>Q1-2: I compensate myself for the time or energy spent by indulging in purchases.<br>Q1-3: I tend to buy non-essential items more frequently or in greater quantities.<br>Strongly disagree = 1, disagree = 2, neutral = 3, agree = 4, strongly agree = 5	2.849	0.937
Green purchasing behavior	GPB	Q2-1: I remind myself to make eco-friendly choices when shopping.<br>Q2-2: I prioritize environmental considerations as a key factor in my purchasing decisions.<br>Q2-3: I feel a responsibility to protect the environment through my consumer choices.<br>Strongly disagree = 1, disagree = 2, neutral = 3, agree = 4, strongly agree = 5	3.900	0.754
Core explanatory variables				
Development-driven overtime	DDO	Q3-1: I work overtime because I enjoy the challenges and sense of accomplishment that work brings.<br>Q3-2: I work overtime because it allows me to enhance my personal skills and professional competence more quickly.<br>Q3-3: To ensure high-quality completion of tasks that interest me, I am willing to dedicate additional working hours.<br>Q3-4: I work overtime to gain opportunities for promotion.<br>Strongly disagree = 1, disagree = 2, neutral = 3, agree = 4, strongly agree = 5	3.077	0.952
Subsistence-driven overtime	SDO	Q4-1: The main reason I work overtime is to earn extra income.<br>Q4-2: Receiving substantial overtime pay is a major incentive for me to put in extra hours.<br>Q4-3: Working overtime to earn monetary rewards helps alleviate my financial burden.<br>Strongly disagree = 1, disagree = 2, neutral = 3, agree = 4, strongly agree = 5	3.233	1.138
Belonging-driven	BDO	Q5-1: I work overtime because I feel attached to the organization.	3.536	0.879

overtime		Q5-2: I work overtime because I regard the organization as my “home.”<br>Q5-3: I work overtime because I feel very comfortable in the organization.<br>Strongly disagree = 1, disagree = 2, neutral = 3, agree = 4, strongly agree = 5		
Escape-type overtime	ETO	Q6-1: Overtime is a great escape route.<br>Q6-2: Working overtime allows me to avoid the tedium of domestic life.<br>Q6-3: Sometimes working overtime allows me to avoid unnecessary social engagements.<br>Strongly disagree = 1, disagree = 2, neutral = 3, agree = 4, strongly agree = 5	2.215	0.798
Peer-pressure overtime	PPO	Q7-1: In my company/department, those who can endure hardship and frequently work overtime are more highly regarded by management.<br>Q7-2: My direct supervisor frequently works overtime, which has created an unspoken pressure for me to do the same.<br>Q7-3: None of my colleagues have left yet, so even if I've finished my tasks, I feel awkward about clocking out on time.<br>Q7-4: In my company/department, overtime is a common occurrence.<br>Strongly disagree = 1, disagree = 2, neutral = 3, agree = 4, strongly agree = 5	3.245	0.937
Mandatory overtime	MO	Q8-1: I work overtime because my supervisor issued a direct and explicit overtime order.<br>Q8-2: I work overtime because the environment around me (or my superiors' attitude) leaves me with no other choice.<br>Q8-3: I often receive urgent tasks before leaving work or on short notice, forcing me to work overtime.<br>Strongly disagree = 1, disagree = 2, neutral = 3, agree = 4, strongly agree = 5	3.394	0.854
Mediating variables				
Perceived Control of Time	PCT	Q9-1: I can manage how I allocate my time in daily life.<br>Q9-2: I can decide when to take my annual leave or personal leave.<br>Q9-3: I can decide when to rest or relax.<br>Strongly disagree = 1, disagree = 2, neutral = 3, agree = 4, strongly agree = 5	3.554	0.856
Perceived Control of	PCF	Q10-1: I rarely consider my budget when shopping.	2.515	0.889

Finance		Q10-2: I don't pay much attention to prices when shopping.<br>Q10-3: My financial situation is relatively comfortable.<br>Strongly disagree = 1, disagree = 2, neutral = 3, agree = 4, strongly agree = 5		
Perceived Psychological Control	PPC	Q11-1: Lately, when I'm feeling down, I find it hard to shake off this mood.<br>Q11-2: I find it hard not to dwell on those troublesome matters.<br>Q11-3: When I feel anxious or tense, it seems like nothing helps me relax.<br>Strongly disagree = 1, disagree = 2, neutral = 3, agree = 4, strongly agree = 5	2.924	1.100
Perceived Behavioral Control	PBC	Q12-1: I believe purchasing eco-friendly products is relatively easy.<br>Q12-2: The initiative to purchase green products lies in my own hands.<br>Q12-3: Whether or not to buy green products is entirely up to me.<br>Strongly disagree = 1, disagree = 2, neutral = 3, agree = 4, strongly agree = 5	4.041	0.644
Perceived Outcome Control	POC	Q13-1: I believe that individual consumers can make a tangible difference in environmental improvement by purchasing green products to participate in pollution control.<br>Q13-2: I believe that individuals' purchasing choices for green products can have a certain impact on pollution control and the conservation of natural resources.<br>Q13-3: I believe that choosing to purchase green products from socially responsible companies can have a positive impact on environmental protection and resource conservation.<br>Strongly disagree = 1, disagree = 2, neutral = 3, agree = 4, strongly agree = 5	4.061	0.613
Moderating variable				
Family Emotional Support	FES	Q14-1: My childhood was very happy.<br>Q14-2: I respect my parents.<br>Q14-3: My parents often argue.<br>Q14-4: My relationship with my mother is pleasant and close.<br>Q14-5: My home is filled with tension and disagreements.<br>Q14-6: I enjoy spending time with my family.<br>Q14-7: My relationship with my father is both pleasant and close.<br>Q14-8: My family listens to and pays attention to what I say.	3.995	0.666

		Q14-9: My parents rarely quarrel.<br>Q14-10: Sometimes physical violence occurs among my family members.<br>Strongly disagree = 1, disagree = 2, neutral = 3, agree = 4, strongly agree = 5		
Organizational Green Climate	OGC	Q15-1: Participating in and supporting green and sustainable development initiatives is important within my organization.<br>Q15-2: My organization is interested in supporting environmental policies.<br>Q15-3: My organization believes that protecting the environment is important.<br>Q15-4: My organization is committed to becoming more environmentally friendly.<br>Q15-5: My organization emphasizes reducing the use of paper, energy, and disposable products.<br>Strongly disagree = 1, disagree = 2, neutral = 3, agree = 4, strongly agree = 5	4.015	0.693
Covariates				
Gender		Q1: What is your gender?<br>Male = 1, Female = 2	1.54	0.498
Age		Q2: What is your age?<br>45 years old and above=1, 41–45 years old=2, 36–40 years old=3, 31–35 years old=4, 26–30 years old=5, 25 years old and below=6	3.87	1.275
Income		Q3: What is your monthly income?<br>Below 2,000 yuan=1, 2,001–5,000 yuan=2, 5,001–10,000 yuan=3, 10,001–20,000 yuan=4, 20000 yuan or above=5	3.38	0.745
Education level		Q4: What is your education level?<br>Junior high school or below=1, Senior high school / technical secondary school=2, Junior college=3, Bachelor's degree=4, Master's degree or above=5	3.88	0.537
